# Feasibility of training practice nurses to deliver a psychosocial intervention within a collaborative care framework for people with depression and long-term conditions

**DOI:** 10.1186/s12912-016-0190-2

**Published:** 2016-12-07

**Authors:** Lisa A. D. Webster, David Ekers, Carolyn A. Chew-Graham

**Affiliations:** 1 School of Social and Health Sciences, Leeds Trinity University, Brownberrie Lane, Horsforth, Leeds, LS18 5HD UK; 2 Wolfson Research Institute for Health and Wellbeing, Durham University/Tees Esk and Wear Valleys NHS Foundation Trust, Queen’s Campus, University Boulevard, Stockton on Tees, TS17 6BH UK; 3 Clinical Academic Training, Research Institute, Primary Care and Health Sciences, Keele University, Keele, Staffordshire ST5 5BG UK

**Keywords:** Collaborative care, Depression, Behavioural activation, Practice nurses, Case managers, Normalisation process theory

## Abstract

**Background:**

Practice nurses (PNs) deliver much of the chronic disease management in primary care and have been highlighted as appropriately placed within the service to manage patients with long-term physical conditions (LTCs) and co-morbid depression.

This nested qualitative evaluation within a service development pilot provided the opportunity to examine the acceptability of a Brief Behavioural Activation (BBA) intervention within a collaborative care framework. Barriers and facilitators to engaging with the intervention from the patient and clinician perspective will be used to guide future service development and research.

**Methods:**

The study was conducted across 8 practices in one Primary Care Trust ^1^ in England. Through purposive sampling professionals (*n* = 10) taking part in the intervention (nurses, GPs and a mental health gateway worker) and patients (*n* = 4) receiving the intervention participated in semi-structured qualitative interviews. Analysis utilised the four Normalisation Process Theory (NPT) concepts of coherence, cognitive participation, collective action and reflexive monitoring to explore the how this intervention could be implemented in practice.

**Results:**

Awareness of depression and the stigma associated with the label of depression meant that, from a patient perspective a PN being available to ‘listen’ was perceived as valuable. Competing practice priorities, perceived lack of time and resources, and lack of engagement by the whole practice team were considered the greatest barriers to the implementation of this intervention in routine primary care.

**Conclusion:**

Lack of understanding of, participation in, and support from the whole practice team in the collaborative care model exacerbated the pressures perceived by PNs. The need for formal supervision of PNs to enable them to undertake the role of case manager for patients with depression and long-term conditions is emphasised.

**Electronic supplementary material:**

The online version of this article (doi:10.1186/s12912-016-0190-2) contains supplementary material, which is available to authorized users.

## Background

Of the total global burden of disease, untreated mental disorders account for 13% [1]. Currently depression is the third leading cause of this burden, affecting approximately 121 million people worldwide, and by 2030 depression is set to rise to become the greatest cause of disease burden globally, overtaking ischaemic heart disease [2]. The clinical and global impact of this is further exacerbated when considered alongside long-term conditions. Thirty per cent of people with a long-term condition have co-morbid mental health problems, of which 20% may suffer from depression [3]. Those people with two or more long-term conditions are seven times more likely to have depression than those without, raising healthcare costs by at least forty-five per cent per person [4] and resulting in significantly greater reductions in healthcare status [5].

Primary care plays an integral role in the diagnosis and management of people with depression particularly when comorbid with a long-term condition [6]. In the UK, 90-95% of patients with depression are treated in primary care [7]; as such its management forms a significant part of the work of primary care services [8]. Yet organisational barriers (e.g., IT systems and multi-site practices) can hinder communications between professionals, particularly when managing patients with more complex problems [9]. Single-disease approaches can lead to a fragmentation of care [10]. The management of depression in people with long-term conditions is further complicated due to the normalisation of distress by both the patient and the practitioner [11]. Evidence has suggested that interventions may be best focused in two areas. Firstly, educational interventions that concentrate on practitioners’ formulation of depression in terms of their ability to conceptualise and verbalise with patients [12], and secondly, enabling collaborative management strategies for depression in long-term conditions by adjusting the structure and organisation of primary care services [13].

Collaborative care is an organisational framework harnessing a multi-professional approach to patient care [14] involving a structured management plan, scheduled patient follow-ups and enhanced inter-professional communications. Evidence from the US and UK supports the use of collaborative care models to improve the quality and access of mental health care [15], as well as enabling the integration of physical and mental healthcare [13, 16, 17].

The UK Medical Research Council [18] has stressed the importance of qualitative evaluations within RCTs in understanding the problems of integrating interventions into healthcare settings with respect to barriers and facilitators of implementation [19]. Nested within the CADET (The Clinical and Cost Effectiveness of Collaborative Care for Depression in UK Primary Care Trial) study [15], Coupe et al. described how although primary care professionals valued the potential for collaboration, GPs’ understanding of collaborative care alongside organisational barriers hindered opportunities for communication. The authors suggest that further work is needed to address these organisational barriers in order to facilitate collaboration around individual patients with depression, including shared information technology systems, facilitating opportunities for informal discussion and building in formal collaboration into the collaborative care framework. A qualitative evaluation within the COINCIDE (Collaborative Interventions for Circulation and Depression) trial [13] indicate that collaborative care can facilitate access to depression care in ways that overcome stigma and enhance the confidence of multidisciplinary health teams to work together [20]. In both the CADET and COINCIDE trials, psychological well-being practitioners delivered the psychosocial intervention to patients, working in collaboration with primary care clinicians.

Practice nurses within primary care have been highlighted as a valuable resource to be utilised and appropriately placed to manage patients with chronic and recurrent depression [21]. Buszewicz et al. [22] demonstrated, within the PROCEED (Pro-active Care and its Evaluation for Enduring Depression) trial, the acceptability of practice nurses (PNs) acting as case managers by patients themselves, but also a reported patients’ unwillingness to discuss their mental health problems with their general practitioner (GP), preferring to talk to the PN. Ekers et al. [23] also suggest that practice nurses are well placed to manage depression in patients with long-term conditions. Evidence from a service evaluation of telephone delivered nurse case management for depression showed it was associated with a mean reduction of 8.9 points on the PHQ-9 (Patient Health Questionnaire 9) five years post-training, moreover a mean change in depression severity was similar in a sub-group analysis of 37 patients with long term-conditions [24].

Behavioural Activation (BA) [25] is a simple psychological therapy for depression, the focus of which is to encourage people to engage with psychologically healthy activities that have been interrupted by changes in life circumstances. Recently it has been shown to be both clinically and cost-effective when compared to cognitive behavioural therapy (CBT) [26]. Not only does it lend itself to dissemination to health care practitioners [27], it can be incorporated into collaborative care approaches [Brief Behavioural Activation in Collaborative Care (BBACC)]. With continuing evidence supporting the role of the practice nurse as case manager, a service development project with a nested qualitative study was conducted in one Primary Care Trust^1^ in North of England with the aim of training practice nurses to deliver a brief behavioural activation (BBA) intervention within a collaborative care framework (BBACC), to patients with depression and one or more long-term health conditions, and evaluate the patients and clinicians perspectives and experiences of receiving and delivering the intervention. For further detail on recruitment to the service evaluation refer to Additional file 1.

Practice nurses (qualified via a Nursing and Midwifery Council (NMC) approved degree in nursing and completed Level 5 of General Practice Nurse Framework) were trained by DE in Brief Behavioural Activation (BBA) in order to deliver the intervention as part of their intended role as case manager, to monitor, support and manage patients with co-morbid depression and long-term conditions in primary care. GPs provided clinical advice and support to the PNs and a mental health specialist (mental health gateway worker (MHGW)) was available (subject to practice resources) to provide support to the PN through discussion of cases, as well advice about sign-posting and referral. Change in depression symptom level and service use were evaluated, a summary of which can be found in Additional file 2.

### Objectives

A nested qualitative evaluation of the service development pilot provided the opportunity to examine the acceptability of the BBA intervention delivered within a collaborative care framework to patients with depression and long-term conditions. Degree of acceptability was evaluated from the patient perspective as well as the practice nurses delivering the intervention, the GPs’ role in supporting the PNs, and the role of the mental health specialist. The overall aim was to examine potential barriers and facilitators to engaging with the intervention from the patient and clinician perspective in order to guide future service development and research in this area.

## Methods

This nested qualitative study received approval from Durham University School of Medicine Pharmacy and Health Ethics Sub-committee (ESC2/2013/13) and NHS approvals were obtained (ref 001_20_11_13_0000). The study was carried out in accordance with the principles outlined in the Declaration of Helsinki. All participants gave informed consent to participate in the study as well as permission for anonymised data to be published.

### Sampling and data collection

#### Recruitment of clinicians

Recruitment of clinicians to the qualitative study was by personal invitation from the research team to those participating practices who took part in the service development project. The invitation was made after 2 months of working within the collaborative care framework in order to explore the implementation of the intervention within the practice.

#### Recruitment of patients

Invitation of patients to participate in an interview was via PNs proving written information sheets about the interview, approximately 2 months after the patient had received the intervention. The information sheet included information on why they are being asked to take part, what will be required of them and the possible advantages/disadvantages of taking part. A consent-to-contact form was provided and if returned to the research team by the patient, the patient was then contacted to discuss the study and arrange a suitable time for interview. Prior to the interview taking place patients returned a signed consent form to the research team. To reimburse participants for their time, £20 love to shop vouchers were offered to those willing to participate.

#### Participant demographics

In this pragmatic study, conducted within one PCT in the North of England, recruitment of clinicians was challenging. This subsequently affected recruitment of PNs and patients. Overall, a sample of ten clinicians was interviewed comprising of 5 GPs, 3 Practice Nurses, 1 Health Care Assistant and 1 Mental Health Specialist. Five patient participants were recruited with four completing the interview.

The demographics of patients and clinicians are reported in Tables 1 and 2 respectively.

**Table 1:** Patient demographics

ID	Gender	Age	LTC Number	LTC reported
PX01	M	53	2	Diabetes, Osteoarthritis
PX02	F	71	3	Rheumatoid arthritis, Diabetes, Hypertension
PX03	F	58	1	low back pain
PX04	F	62	1	Diabetes

**Table 2:** Clinician demographics

ID	Role	Gender	Practice size^a^
GP01	GP	F	11396
GP02	GP	F	8401
GP03	GP	M	25386
GP04	GP	M	4402
GP05	GP	F	12678
PN01	Practice Nurse	F	11396
PN02	Practice Nurse	F	8401
PN03	Practice Nurse	F	25386
HCA	HCA	F	4402
MHGW	Mental Health Gateway Worker	F	25386

^a^Figures retrieved from NHS UK October 2015

#### Interviews

Topic guides were developed by the research team based on the literature in this area; for patients we aimed to explore the acceptability of the intervention delivered by PNs; topic guides for PNs, GPs and MH Specialists explored facilitators and barriers to delivery of the intervention within primary care. The researcher (LW) conducting the interviews was an experienced post-doctoral researcher and supported by CCG and DE.

#### Clinicians

Semi-structured interviews of clinicians were conducted face-to-face at the practices once written consent was obtained. All interviews were digitally recorded with consent. Specific topics for clinicians included their understanding of collaborative care, clinician involvement in its implementation, and how their work is organised and delivered under the collaborative care framework.

#### Patients

Semi-structured interviews for patients were conducted over the telephone and digitally recorded with consent. Love to shop vouchers in the amount of £20 were offered for those willing to participate to reimburse them for their time.

Topics for patient interviews included prompts about understanding of depression and experiences of discussing symptoms with and receiving help from PNs.

#### Analysis

Interviews were transcribed verbatim, the transcripts forming the data for analysis. Data were coded and analysed by LW and CCG separately then discussed in more detail to agree upon emergent themes. Themes were compared within data sets (clinicians/patients) as well as across data sets to gain a detailed understanding of patient experiences, and if and how the intervention within the collaborative care framework, added to the clinicians’ perspective of the management of people with depression and long-term conditions. Initial analysis involved a thematic approach using the principles of constant comparison [28], with further analysis using Normalisation Process Theory (NPT) [29].

This specific approach was employed to aid in the analysis process as it provides a conceptual framework for understanding and evaluating the workability and sustainability of complex interventions, focusing on the routinisation of intervention components [29]. NPT has been successfully employed to guide evaluations of implementation of depression care in health care settings.

NPT focuses on four theoretical constructs through which implementation can be understood:

- Coherence: The meaning of the practice to participants
- Cognitive participation: engagement, individually and collectively, with the practice
- Collective action: Interaction with pre-existing or established processes
- Reflexive monitoring: How the practice is assessed and understood by the participants.

Examining the extent to which an intervention can become integrated within everyday practice informs the degree of sustainability of the intervention.

The advantages of utilising NPT are that it can be used to address the feasibility of implementation, with the NPT framework aiding in recognising which components of implementation may pose particular barriers.

## Results

### Initial thematic analysis

Five main themes emerged from the initial thematic analysis (see Table 3 for illustrative data): (1) Awareness of depression, (2) Time/someone to listen, (3) Stigma, (4) Up-skilling of PNs and (5) Competing practice priorities. Illustrative data is given, with identifiers.

**Table 3:** Initial thematic analysis

Salient themes	Key elements	Illustrative data
Awareness of depression	Levels of understanding from the patients about how they are feeling and why.	What I couldn’t understand, love, is that I like to think I’m probably above average intelligence. I’m not a thicky, I’m not a thicko, and I just can’t understand why I feel like I do when I shouldn’t be doing. (PX01)
Time/someone to listen	From a patient perspective time is crucial in affording them to open up to a professional and feel like they are being listened to.	Yeah, I think the time factor is a lot to do with it. You know, you can just open up and speak and she’ll listen and advise, you know, where doctors haven’t really got that time with you, (PX02)
Stigma	Patients may feel more comfortable disclosing their physical health problems compared to their mental health problems to professionals.	I don’t worry about my physical health; it’s the mental health I dread really. I would much rather have a physical problem than a mental problem, because it’s horrible. My ex-husband or my, he’s passed away now, but he used to be, it was a stigma to him if you can understand. And I used to say if you could just have half-an-hour of how I feel you would know. People don’t understand it unless they’ve had it. (PX02)
	Stigma from professionals who do not have the skills or confidence to approach patients with mental health concerns	There are barriers and some of them are from the patient and some of them are from the health professional, because a lot of health professionals don’t feel confident about talking about mental health issues to patients. They feel they can’t say the S word, the suicide, you know if people have been suicidal in the past they feel that’s a difficult conversation to have, and that’s all about education isn’t it really and collaboration and trying to make connections between agencies that could support you in doing those sort of things, but you’re up against it a little bit I think (PN02)
Up-skilling of PNs	The up-skilling of PNs leads to an increase in their confidence and abilities in addressing mental health concerns with their patients. However, this can only be achieved through adequate and effective supervision.	I think having a GP on the premises throughout the day, I think that was like a mental support for me as well. So if somebody said something that I was concerned about I could go and speak to the GP rather than leaving a message or picking up the phone or something. So I think that was like a strong point for me mentally, I felt that there is somebody that I can turn to straightaway if there is a problem. (HCA)
Competing practice priorities	Resource issues lead to competing practice priorities for different practice staff. This can result in the implementation of tick-box exercises for those lower priorities.	To actually coordinate it and to put all the information on there, and then obviously getting the patients onto the programme and then monitoring them and reviewing them regularly and having that formal communication, because you’ve got to understand that you’ve got so many competing priorities. (GP04)

Initial thematic analysis and illustrative data is summarised in Table 3.

Following initial thematic analysis the data were analysed using the framework of the four NPT concepts of coherence, cognitive participation, collective action and reflexive monitoring to explore how this intervention could be implemented in routine practice.

#### Coherence

Understanding the collaborative care framework.

The psychosocial intervention within this collaborative care study was Brief Behavioural Activation for Collaborative Care (BBACC). GPs reported that they found the simplicity of the intervention attractive:

“*In essence it’s quite simple really when you boil it down, if you can get people to go out and do things and be part of the community again and not isolate themselves they feel better. It’s not rocket science, it’s getting them to do it is difficult, but the concept itself is very simple. And that appeals to me because a lot of therapies they get so complicated and so navel gazing and you just think well -…..- where are you going with this, you’re just making the person dwell on all their problems” (GP02).*

In addition, BA was perceived to integrate easily into the long-term conditions management:

*“Actually this is quite a practical way of getting involved in something while you’re actually doing their long-term conditions management. So for me I can see it’s a very practical based therapy”* (GP01)*.*

The understanding and subsequent delivery of collaborative care varied amongst GPs and practices:

“*It’s not something that I've read an awful lot about”* (GP03) to “*collaborative care is another word for integrated care*” (GP01).

Furthermore, the delivery of collaborative care within the pilot was seen to vary across practices:

“*In terms of collaborative care, you’ll probably find some practices will always be better than others at delivering that”* (GP02).

The role of the practice nurse was seen to be integral to the collaborative care process:

*“I think they’re probably best suited to be case manager from that point of view, because they’ve got the relationship, they’re seeing the patient regularly anyway from a chronic disease point of view, and it leads to more holistic and joined up care, rather than patients having to go to different practitioners for different parts of their health”* (GP03).

Although there was concern expressed that *“you can create a dependency”* (GP01) on the nurses, it was felt that a patient’s preference would be to see a nurse as opposed to mental health professional outside of their practice:

“*It’s very much more acceptable for our patients to see someone regarding mood at the practice as opposed to going externally to see a counsellor. I think it’s much more acceptable when it’s seen to be the nurse, or the diabetic nurse. People like to hang hooks on names, patients don’t generally go round talking about their depression, but you do hear them going around all the time talking about their diabetes or their angina or whatever”* (GP05).

The MHGW also regarded the practice nurse as suitable to deliver the intervention as their role is more supportive in nature:

“*because I think sometimes they don’t particularly need a specialist input, but just need some help and support to get back on track after, particularly after quite a bit diagnosis for them really”* (MHGW).

Although PNs were regarded as best placed to deliver the intervention by GPs and the MHGW, they themselves perceived that their role may inadvertently act as a barrier to care initially because patients *“… just perceive me in a certain role and I wear a uniform as well, and I didn’t know whether that put a barrier up a little bit for some of them that they had this perspective of me just delivering their management for their disease and why on earth is she talking to me about how have I been feeling, down, depressed, hopeless,..”* (PN02)*.*

#### Cognitive participation

Establishing relationships within the collaborative care framework, engaging participants to invest in the new way of working and identifying barriers to engagement.

For the process of collaborative care to be effective, all participants within the framework must be fully engaged with the process. From a GP perspective, data suggests that there needs to be investment from the whole practice:

*“Well I suppose the other thing was that obviously I had to take it to a practice meeting, because it impinged on (names practice nurse) doing other work for the practice. Because we knew it was going to take quite a bit of a time. So we run it past the rest of them. If I’m honest I don’t think the rest of them were that interested, which is always a problem in a practice. So they knew it was going on, but they weren’t really actively part of it and didn’t get involved” (GP02).*

Having a “dedicated GP partner who’s interested in mental health to help sort of champion the project” (GP01) was suggested to facilitate the new way of working, however, barriers exist when “*we’re trying to coordinate with our nurses and get feedback on things, when you’re trying to support them through things, collaborating is difficult because of time to meet together” (GP05).*

Practice Nurses suggested that this way of working is standard for them, the only difference being their confidence levels have increased with regard to addressing patient’s mental health:

“*Well, it shouldn’t be any different really should it, and I have to say since I’ve been doing this there is a bit more of holistic-ness about and I’ve got more confidence about talking to people and about the mental health issues” (PN02).*

Patients identified time as a barrier to access to care, however only with regard to disclosing problems to GPs. PNs were perceived to have more time to listen, whereas GPs are more limited:

*“Yeah, I think the time factor is a lot to do with it. You know, you can just open up and speak and she’ll listen and advise, you know, where doctors haven’t really got that time with you” (PX02).*

Stigma also featured as a barrier to accessing care for depression as it inhibits the engagement process. However, this could be overcome through the process of BBACC:

*“So they might come in that they’re not sleeping, they’re tired, they’re fed up, and I can imagine it would take three or four more visits to get to the bottom of what it is. So it could be all of them symptoms are low mood, but the way society is they don’t want to say I am depressed, please put me on an antidepressant, so there was a stigma around it”* (PN03).

This was reinforced by patients themselves:

*“Well I get embarrassed anyway whoever I speak to. After the initial first meeting I felt a bit more at ease with her you know” (PX03)*

Patient data consistently stressed however, that it is more about gaining understanding about depression and what contributes to it:

*“..obviously you don’t realise it yourself but it does lead to these sort of things, you being tired all the time because you’re always in pain. But you don’t look at it that way, well I certainly didn’t anyway until she explained it to me”* (PX01).

#### Collective action

How will working within a collaborative care framework affect participants and how they behave?

GPs recognised that providing this service within the practice had the potential to benefit the patients for several reasons. GPs suggested that patients were able to speak to a person they were already familiar with:

*“Well I think patients benefited because I think they saw somebody that they were familiar with. They hopefully had somebody that they had confidence in, that they could articulate their concerns, that they felt that their problems were being taken seriously, and that they were able to come to some shared goals, so I think the whole idea is that using (names PN) as somebody who they developed a trusting relationship with” (GP04)*

This was also recognised by the PNs and patients:

*“Yeah, I think so, because they’re not having to think about going somewhere else, [identifies area], and I think the doctors and that know them here, so yeah. And they haven’t got such a long waiting time, I mean with me I can normally get them in the next week, and my clinic’s not always full” (PN03).*

Patients explained that it is not only the location of the service that enables patients to engage, but also familiarity with the PNs:

*“I think it’s handy to have somebody like that. You know, I mean I used to go to a counsellor after I came out of hospital, that’s 20 years ago now, I used to have to go to a counsellor, but when it’s up at your own doctor’s surgery it’s much easier to do that”. (PX02)*

*“My GP offered me counselling through another party, and I rejected it. Because I feel comfortable with (names PN) you know what I mean.” (PX04)*

GPs suggested that participation in the pilot has led to up-skilling of the PNs and an increase in their confidence in dealing with patients with MH problems:

*“I think also the mind-set as well has probably changed because when she’s actually seeing patients now she’s probably looking at some things a little bit differently than she was previously. So hopefully there should be some real added learning that if there are patients there who are distressed, who are worried and everything else, that she will have a clear understanding of how she can navigate their care and it wouldn’t just be about taking their blood pressure or pulse or whatever”. (GP04)*

PNs also reported continued use of these skills in their day to day practice:

*“Initially it was really helpful having all the information that we got from you, because it was a bit of a crib sheet and it was great for prompting and focusing the conversations.” (PN02)*

Patients also reported the continuing use of techniques from the intervention to help them in their everyday lives:

*“If I go into sort of situations remember what she’d been talking about and such, things like that. And I did, because I’m terrible, I get terrible road rage, and that’s not half as bad as it used to be because obviously you think about what she’s said and you try and assess the situation. Well I do anyway, that’s what it’s made me do.” (PX01)*

Furthermore, GPs reported that participation had focused the efforts of the practice as a whole to look beyond a patients’ long-term condition as well as integrating more with other service providers in the local area:

*“So I think overall I think it has in a way that the focus for the practice has not just been on management of long-term conditions but it’s also been looking at patients with mental health and how we can help them. As a by-product of this I think that we did a little bit more work on finding out what voluntary community sector groups were out there as well”. (GP04)*

Although GPs noted that participation in this service development might have led to an increase in skill and confidence of the PNs, PNs themselves felt it impacted upon themselves negatively with respect to their own psychological health:

*“And if you’ve only got ten minutes and these are patients who often sit down and cry in front of you and you can’t get them out very easily in just ten minutes, so it’s quite a drain on me as well psychologically” (PN01).*

*“It’s been a good experience, but like I said it’s also been very challenging, and I think you, when you’re dealing with these sort of patients I don’t know whether I was new to this, I found it really challenging and felt really drained at the end of it, with a headache and sort of feeling have I done enough for them, did I do it right?” (HCA).*

This was reported to be partially mitigated by the support available from the GPs and MHGWs:

*“So if there was an issue she could come and speak to me about it. And I offered if she wanted to just generally talk about her caseload then she could come, or if she wasn’t sure about something then she could come and talk to us about it” (MHGW).*

However, this was not the case for all practices:

*“some of the GPs, I work three days but some of the locums now I don’t even know what they look like never mind knowing them, and I think it’s a lot easier if you know them and have that face to face contact” (PN01).*

Thus the key barrier to implementation of the collaborative care model reported by GPs and PNs was time for the professionals involved. For the GPs, time to coordinate support through supervision was suggested to be a barrier. From the PN perspective, most found the level of support from the GP and MHGW sufficient simply by being on the same premises. When this was not the case support appeared to be lacking. GPs, PNs and patients all stated that being able to deliver/receive the intervention at their own practice was key to engaging the patient as well as focusing the efforts of the professionals both within and external to the practice.

#### Reflexive monitoring

Evaluating the collaborative care framework, identifying barriers to sustainability.

GPs and PNs acknowledged that the main contributory factor to these barriers is the overarching issue of resources available to practices:

*“So I’m afraid what we have to concentrate on these days is how we get the money into the practice to pay people, because we’re a business at the end of the day. So whereas as this is a nice to do, and I would love my practice nurse to be able to do it, I’m afraid practically it’s just not going to be possible, not without funding and I can’t see that happening”. (GP02)*

PNs also noted difficulties with the intervention not being a priority for the practice as a whole, thereby negatively impacting on the process of collaborative care itself:

*“We were supposed to use a PLT, which is the Practice Learning Time on a Thursday afternoon, to feedback to the larger group and that never happened either, because it kept getting relegated because there were rather more important topics that kept getting pushed to the front.” (PN01)*

Engagement and recruitment were also impacted by practice priorities. This required high levels of communication from the PN to avoid disengagement from participants and resultant drops in recruitment levels:

*“It’s a difficult thing to keep uppermost in people’s minds if you’re not constantly saying to them, don’t forget, don’t forget, don’t forget about BAT, you know, BA, because there’s all sorts of other things going on.” (PN02)*

With regard to the sustainability of the intervention, GPs differed in their opinion of how this could be achieved:

*“So yes I think it is something worth doing, it’s just who would provide it. It may be more beneficial, and more successful, if it was provided by say a gateway worker or somebody like that within the practice, rather than practice nurses” (GP01).*

Although both patient and PN participants reported a variety of benefits stemming from the service provided, barriers to engagement and recruitment existed because of a lack of collaborative working within practices as a whole. Furthermore, overarching resource issues meant that practice priorities were constantly changing, capacity reducing, and therefore the ability to provide such services despite the advantages, was challenging.

## Discussion

### Summary of main findings

This qualitative study, used to evaluate a pilot service development, suggests that both GPs and PNs accepted collaborative care as a coherent framework, and found the intervention acceptable in terms of its simplicity and its workability within long-term condition management. PNs were regarded as key to the process of collaborative care and suitably placed to deliver the intervention due to their role within the practice, working with patients with long-term conditions.

GPs’ understanding of the collaborative care framework however, was limited, thereby impacting on participation in the pilot. Despite PNs attempts to employ a collaborative care approach, the perceived lack of understanding and participation from GPs led to PNs suggesting the need for a dedicated GP who can facilitate the necessary investment from the practice.

Both GPs and PNs reported the positive impact the training in BBA intervention had on the PNs with respect to their skills and confidence in addressing mental health problems with patients. However, PNs recounted the negative impact it has had on their own psychological and mental wellbeing, emphasising the need for supervision and support for PNs if they are to take on this additional role.

Results suggest that practice priorities and available resources pose the greatest barrier to the sustainability and therefore feasibility of such interventions. Lack of participation and/or understanding from GPs in the collaborative process further exacerbate the pressures placed on PNs, leading to the question of whether PNs are best placed to undertake the role of case manager.

### Comparison with the existing literature

It is suggested that PNs are best placed to manage depression in people with long-term conditions [23]. Our study reports positive findings regarding the up-skilling of PNs and increased confidence levels when dealing with mental health problems. As reported by Bennett and colleagues [21], patients also described feeling more comfortable opening up to PNs compared to GPs as they had more time to talk. It would appear then that the need for external mental health providers to come into primary care is no more, as the integration of mental and physical healthcare can be facilitated through the PNs. However, the feasibility and sustainability of nurse-led collaborative care for depression and long-term conditions has recently been called into question [20].

Previous qualitative work with nurses who are responsible for the delivery of psychological interventions for patients with long-term conditions has reported the challenging nature of the work [30]. In the current study, PNs describe a negative impact on their emotional wellbeing, when working with patients with complex MH problems. This is also reflected in an updated service evaluation of telephone delivered nurse case management for depression. Of the 13 nurses that were trained in the collaborative care service evaluation, only 3 continued to do so. Those who ceased delivering the intervention did so for several reasons including time/staffing issues, financial support and the negative emotional impact the intervention had on the PNs. Furthermore, the lack of regular supervision for PNs was suggested to amplify the stresses experienced [24].

The value of enhanced supervision of case managers within the collaborative care framework has been highlighted previously [31] and suggested as a means to facilitating professional collaborations as well as improving GPs’ understanding of the framework. Within the current study, supervision was available but levels of support accessed differed across practices, dependent on the location and time constraints of the GP. Supervision was also available from a MH specialist, however this existed for only the one practice whose MH specialist signed up for the study.

Such organisational barriers could be overcome through ensuring the provision of more systematic and enhanced supervision. Moreover, the sharing of information in the absence of co-location may also help facilitate the necessary level of professional collaboration.

### Strengths and limitations of the study

The strengths of this study lie in its multi-perspective approach. Analysis of clinician and patients’ perspectives adds to the existing evidence-base [11, 20, 21] and draws attention to the perceived barriers and facilitators to efficient patient care. Furthermore, utilising NPT via the four constructs (coherence, cognitive participation, collective action and reflexive monitoring) adds to the rationale of using this method to analyse the normalisation of complex interventions within healthcare.

This study was a pragmatic study carried out in one PCT in Northern England, and recruitment of clinicians and consequently, PNs and patients was challenging. Recruitment of patients in particular proved difficult despite the offer of reimbursement for participation. Furthermore, resource issues meant that PNs were responsible for the recruitment of patients and data entry in addition to their existing workload.

## Conclusions

The benefit of multi-perspective data such as in the current study, is gaining a greater understanding of the barriers and enablers to the implementation and sustainability of an intervention as perceived by the patients and the providers. The role of the PN from the patient perspective was considered valuable by the patients, however the PNs reported a negative impact on their own emotional wellbeing. It has been suggested that collaborative care for depression and long-term conditions would be better delivered by a coordinated and well supervised team of experts in mental and physical health than by PNs alone [20], which may serve to reduce the burden placed upon the PNs. However, in order for such an intervention to become embedded in routine practice it needs to fall within the constraints of general practice. Since recent research has shown Behavioural Activation to be cost-effective when delivered by junior mental health workers [26], future research would be best focused on a) disseminating such training more widely and b) overcoming organisational barriers and facilitating higher levels of professional collaboration to ensure the feasibility of similar novel interventions.

## Additional files

Additional file 1: Recruitment to service evaluation. Further details of recruitment to the service evaluation, including description of roles. (PDF 191 kb)
Additional file 2: Summary of change in depression symptom level and service use. Tabulated data of average PHQ-9 scores per practice (baseline and post-treatment) and average number of contacts and related costs per practice and patient. (PDF 63 kb)

## Abbreviations

BA: Behavioural activation
BBA: Brief behavioural activation
BBACC: Brief behavioural activation collaborative care
CBT: Cognitive behavioural therapy
CCG: Clinical Commissioning Group
GP: General practitioner
MHGW: Mental health gateway worker
NPT: Normalisation process theory
PHQ-9: Patient health questionnaire -9
PN: Practice nurse

## Footnotes

1 Following changes to the NHS brought about by the Health and Social Care Act 2012, Primary Care Trusts (PCTs) ceased to exist on 31st March 2013. Responsibilities of Bradford and Airedale PCT now rest with Clinical Commissioning Groups (CCGs), which include Airedale, Wharfedale and Craven CCG, Bradford City CCG and Bradford Districts CCG.
