# Supplementary material for: Feasibility of training practice nurses to deliver a psychosocial intervention within a collaborative care framework for people with depression and long-term conditions
Source: BMC Nurs. 2016 Dec 7;15:71. doi: 10.1186/s12912-016-0190-2 (PMC5142437; doi:10.1186/s12912-016-0190-2)
Supplement: Additional file 1: — Recruitment to service evaluation. Further details of recruitment to the service evaluation, including description of roles. (PDF 191 kb) [file 12912_2016_190_MOESM1_ESM.pdf]

Recruitment to service evaluation

Practices were invited to register an interest in the pilot with Bradford and Airedale PCT and commit to identification of at least one practice nurse (or equivalent role) who would take part and build depression case management into LTC clinics currently in operation. Each practice indicated they would allocate 0.5 clinical sessions weekly for the identified practice nurse to be funded by the CCG. Practices also agreed to identify one or more GP's who would be designated with the role of providing clinical advice and supervision to the nurse in relation to this role as well as one administration person to liaise with the evaluation team regarding the collection of anonymous clinical data. In addition, it was deemed to be beneficial to attempt to identify a local MH specialist to support the project and provide a link to local MH services.

Specific roles within defined within the pilot were:

- Practice nurses: To attend training and deliver Brief Behavioural Activation (BBA) led case management as part of their LTC clinic work. Collect session data.
- GP: To provide clinical advice and support to the practice nurse relating to the administration of depression treatments as part of collaborative care.
- MH specialist: To provide support to practices in the setting up and running of the service by offering consultation on cases and access to referral pathways where needed (following consultation as part of CC process).
- Administrative staff: To collate information in consultation with evaluation team and supply this data at agreed time points.
- Pilot Evaluation team: To deliver training and monitor progress of the pilot service evaluation, in addition to problem solving and evaluation of outcomes.

24
