# Supplementary material for: Feasibility of training practice nurses to deliver a psychosocial intervention within a collaborative care framework for people with depression and long-term conditions
Source: BMC Nurs. 2016 Dec 7;15:71. doi: 10.1186/s12912-016-0190-2 (PMC5142437; doi:10.1186/s12912-016-0190-2)
Supplement: Additional file 2: — Summary of change in depression symptom level and service use. Tabulated data of average PHQ-9 scores per practice (baseline and post-treatment) and average number of contacts and related costs per practice and patient. (PDF 63 kb) [file 12912_2016_190_MOESM2_ESM.pdf]

Summary of change in depression symptom level and service use

Table 1: Average PHQ9 per practice at baseline and post-treatment.

Practice	PHQ9 (n=22)	
	Baseline	Post*
A	14.0	7.7
B	16.6	13.9
C	12.0	3.0
D	11.5	7.5
E	No data received	
**F**		
**G**		
**H**		

*last observation recorded

Table 2: Average number of contacts and related cost per practice and patient.

Practice	Total N Nurse Contacts per Practice	Average N Nurse Contacts per Patient	Total Cost per Practice £	Average Cost per Patient £
A	41	3.7	561.70	51.06
B	16	2.0	219.20	27.40
C	8	8.0	109.60	109.60
D	6	3.0	82.20	41.10

E	No data received			
F				
G				
H				
Total	71	3.23	972.70	44.21
